# Granulin as an important immune molecule involved in lamprey tissue repair and regeneration by promoting cell proliferation and migration

**DOI:** 10.1186/s11658-022-00360-6

**Published:** 2022-07-30

**Authors:** Ruixiang Sun, Dong Wang, Yuxuan Song, Qingwei Li, Peng Su, Yue Pang

**Affiliations:** 1 College of Life Sciences, Liaoning Normal University, Dalian, 116081 China; 2 Lamprey Research Center, Liaoning Normal University, Dalian, 116081 China; 3 Collaborative Innovation Center of Seafood Deep Processing, Dalian Polytechnic University, Dalian, 116034 China

**Keywords:** PGRN, Lamprey, Immune response, Tissue repair

## Abstract

Progranulin (PGRN) is an autocrine growth factor that regulates cell proliferation, migration, wound healing, and tissue repair in mammals. Lamprey is the most primitive of the extant vertebrates and is regarded as the survivor of a once flourishing group of paleozoic vertebrates, with a history of more than 500 million years. To date, the evolutionary dynamics and the underlying function of the PGRNs remain largely unclear in lamprey. Here, we screened four genes encoding PGRNs from the genomes of *Lethenteron reissneri* and *Petromyzon marinus*, including one long form (named Lr-PGRN-L) and three short forms (named Lr-PGRN-S1, Lr-PGRN-S2, and Lr-PGRN-S3), and performed phylogenetic tree, functional domain, and synteny analyses to identify the evolutionary history of the four Lr-PGRNs. In addition, the expressions of the four *Lr-pgrn* family genes and the immune response against various pathogenic challenges were also investigated. We found that these genes were widely distributed in various tissues of lamprey and performed a variety of functions. Moreover, our results suggest that Lr-PGRN-S1 induces cell migration and proliferation, and is involved in repair after skin and spinal cord injury under appropriate conditions. Our findings are valuable because they improve the understanding of the evolutionary relationship of vertebrate *pgrn* genes, as well as providing new insights into the diverse and important roles of Lr-PGRNs.

**Supplementary Information:**

The online version contains supplementary material available at 10.1186/s11658-022-00360-6.

## Introduction

Progranulin (PGRN), also called granulin epithelin precursor (GEP), is an autocrine growth factor [1, 2]. In humans, *pgrn* is located on chromosome 17 at cytogenetic band 17q21 and contains 12 exons [3] and codes for a 593-amino-acid 68.5-kDa protein [4]. It includes 7.5 copies of a cysteine-rich domain (named GRN), viz. GRNA, GRNB, GRNC, GRND, GRNE, GRNF, GRNG, and GRNP, in which P is the half domain and A–G are full domains [5]. PGRN is mainly distributed in nerve, immune, and epithelial cells [6, 7]. In previous studies, the ability of PGRN to regulate cell proliferation [6, 8], migration [9], and wound healing and tissue repair [10] has been well characterized. PGRN can induce activation of signal pathways crucial to cell proliferation, such as the phosphatidylinositol-3 kinase (PI3K) and extracellular signal-regulated kinase (ERK) pathways [11, 12]. The activated pathways then induce expression of cyclin D1 and cyclin B, cellular responses that are consistent with its proposed role as a growth factor [11, 12]. A previous study suggested a role of PGRN in bladder tumor progression by promoting the growth and migration of bladder cancer cells [13]. PGRN has also been considered to participate in the cellular response to wounding as both a locally induced growth factor and a paracrine mediator [10]. Interestingly, several lines of evidence have demonstrated that, in the central nervous system (CNS), the expression of PGRN increases significantly upon activation in microglia whereas it increases during maturation in neurons [14]. Moreover, PGRN participates in the regulation of inflammation [9]. Neutrophil elastase secreted by macrophages can cleave progranulin to release GRN peptides, while secretory leukocyte protease inhibitor (SLPI) can prevent the proteolytic conversion of PGRN to GRNs. PGRN and some GRN peptides have been shown to possess opposing inflammatory functions [15, 16].

PGRNs are distributed across the animal kingdom from premetazoans to mammals [17]. Additionally, the evolution and structure of progranulins, as well as granulin domains, have been investigated [18]. In teleost, PGRN was secreted in cell culture supernatants and modulated cell proliferation at various stages of myeloid differentiation [19]. A granulin-like polypeptide derived from *Ciona savignyi* shows high homology with human GRNA, GRNB, and GRNC [20]. Research has revealed that the granulin-like polypeptide significantly inhibited the differentiation and proliferation of cancer cells in vitro. Granulin-like molecules have been identified in *Aedes albopictus* and *Manducasexta* and are functionally conserved between insects and lower vertebrates [21]. Furthermore, parasitic helminths probably use PGRN to modulate cell proliferation and differentiation in vivo. *Opisthorchis viverrini* PGRN induces proliferation of fibroblasts [22].

PGRNs have been widely studied, but their physiological functions and mechanism of action in jawless vertebrates remain largely unexplored. Lamprey and hagfish belong to Cyclostomata and are living representatives of ancient jawless vertebrates [23–25]. Lamprey is a critical organism that provides insights into understanding the phylogeny of early vertebrates and is a very useful model for studying immunology, comparative genomics, cytobiology, developmental biology, etc. [26–28]. In this study, we identified and characterized the evolutionary dynamics of four paralogs of PGRNs, namely Lr-PGRN-L, Lr-PGRN-S1, Lr-PGRN-S2, and Lr-PGRN-S3, in lamprey. Analyses of protein and RNA expression levels revealed that *Lr-pgrn* genes were widely expressed in the tissues of lamprey. Moreover, Lr-PGRN-S1 participated in the proliferation and differentiation of cells. In addition, qRT-PCR showed that the expression levels of *Lr-pgrn* messenger RNA (mRNA) increased significantly after induction by various pathogens, revealing its roles in immunity. Together, these findings revealed that Lr-PGRNs act as chemokine or immune regulator molecules in lamprey and induce growth and development of cells. This research could shed light on the evolutionary dynamics of *Lr-pgrn* genes and lay the foundations for investigating the roles of PGRNs in cell proliferation and the immune response of vertebrates.

## Materials and methods

### Animals and immunization

We caught adult lamprey (*Lethenteron reissneri*) from Tong River, China. The lampreys were then kept in an aquarium at 4 °C. This study was approved by the Animal Welfare and Research Ethics Committee (permit no. SYXK2004-0029). Animal experiments were conducted at Dalian Medical University. Three lampreys were injected intraperitoneally with phosphate-buffered saline (PBS) (0.1 mL for each lamprey), *Staphylococcus aureus*, and *Vibrio anguilarum* (1 × 10^8^ for each lamprey) in each group.

### Cloning and analyzing the open reading frame (ORF) of the Lr-*pgrn-s1* gene

The complementary DNA (cDNA) sequence of the Lr-*pgrn-s1* gene was obtained from the database established in our laboratory. Basic Local Alignment Search Tool X (BLASTX) software was used to confirm the sequence. Total RNA was extracted from lamprey liver tissues. Total RNA was mixed with RNase-free water and stored at −80 °C. Subsequently, we used the PrimeScript II first-strand cDNA synthesis kit (TaKaRa, Dalian, China) to synthesize cDNA as per manufacturer’s instructions. Specific PCR primers for ORF sequences of *Lr-pgrns* were designed using PRIMER 5. The recombinant plasmid was constructed by engineering ORF sequences of *Lr-pgrns* in the pMD19-T simple vector for sequence confirmation. We then used Sequencher 4.2 software to identify the sequences. Moreover, the protein sequences were analyzed using DNASTAR 5.0 and DNAMAN V6 software. These sequence data were uploaded to the GenBank databases under accession numbers OM459826, OM459827, OM459828, and OM459829.

### Phylogenetic tree and genomic synteny construction

Sequences of PGRN molecules of other organisms were downloaded from the National Center for Biotechnology Information (NCBI) database. The multisequence alignments of PGRNs were processed using Bioedit 7.0 software. MEGA 7.0 software was then used to establish the neighbor-joining (NJ) trees depending upon pairwise deletion, which includes a Poisson model matrix of an amino acid model and missing data with 1000 bootstrapped replicates. Genomic synteny was mapped based on data from Genomics and Stowers Institute (http://www.genomicus.biologie.ens.fr. SIMRbase, https://genomes.stowers.org/).

### Preparation of Lr-PGRN-S1 recombinant protein

The ORF sequence of Lr-*pgrn-s1* was inserted into the pCold I vector. Expression of recombinant protein was induced with 0.1 mM isopropyl-β-d-thiogalactoside (IPTG) for 24 h at 16 °C in *Escherichia coli* BL21 (DE3). Then, the expressed protein was purified using Ni affinity chromatography (GE Healthcare) and quantified using a bicinchoninic acid (BCA) protein quantitation kit (Solarbio, Beijing, China). Finally, it was analyzed by sodium dodecyl sulfate (SDS) polyacrylamide gel electrophoresis (PAGE).

### Production of anti-Lr-PGRN-S1 polyclonal antibodies

To raise polyclonal antibodies against rLr-PGRN-S1, New Zealand rabbits were immunized with rLr-PGRN-S1 protein through multipoint intradermal injections four times per 2 weeks. rLr-PGRN-S1 (500 mg) antigen and PBS (500 mL) were mixed using Freund’s complete adjuvant (Sigma) with the same volume at first immunization. For the rest of the immunizations, Freund’s complete adjuvant was changed for Freund’s incomplete adjuvant and the dose of antigen was halved. The antiserum was collected from the rabbits, and the polyclonal antibodies were purified with affinity chromatography using a protein G resin (GE Healthcare). The antibody’s titer was then measured by enzyme-linked immunosorbent assay (ELISA).

### Western blot analysis

SB cells were extracted under 0.25% trypsin treatment from lamprey; other cells and tissues were acquired in the same manner. Radioimmunoprecipitation assay (RIPA) lysis buffer was used for protein extraction. The protein concentration was measured first, then the proteins were analyzed using SDS-PAGE. Next, the sample was diverted to polyvinylidene difluoride (PVDF) membranes. PBS-T solution was used to prepare 5% skim milk powder for PVDF membrane blocking for 3 h. Then, the membrane were incubated with rabbit anti-RLR-PGRN-S1 and anti-L-LECT antibodies at 4 °C overnight. After washing three times for 10 min each using PBS-T, the membranes were incubated with horseradish peroxidase (HRP)-conjugated goat anti-rabbit IgG (1:5000). The membrane was processed by enhanced chemiluminescence (ECL) (Illuminant, Beyotime, China) after washing three times using PBS-T.

### Immunohistochemical staining

Each lamprey tissue specimen was fixed in 4% paraformaldehyde and embedded in paraffin. The sections were deparaffinized using xylene and rehydrated by washing in descending concentrations of ethanol at about pH 7 PBS for 5 min, dried with cold air, and put into a wet box. Endogenous peroxidase was blocked using 3% H_2_O_2_ in methanol for 20 min at about 25 °C. The sections were then blocked by incubating in 10% normal goat serum for 3 h at 25 °C and incubated in anti-rLr-PGRN-S1 rabbit polyclonal antibody as primary antibody. Subsequently, they were incubated in HRP-conjugated goat anti-rabbit IgG (1:100) as secondary antibody. Normal rabbit immunoglobulin G (IgG) was used as negative control. The sections were stained with diaminobenzidine (DAB), with the staining time determined based on the real-time image. After dehydration using an alcohol gradient, the slices were treated with xylene twice for 15 min each and finally mounted with neutral resin.

### Confocal microscopy

Lamprey SB, leukocytes, and liver cells were collected by trypsin treatment. Subsequently, cells were cultured in wells for 25 min with 4% paraformaldehyde at 25 °C. They were then rinsed two times using PBS, fixed, and permeabilized using 0.1% Triton X-100 for 10 min. The cells were then incubated in normal goat serum for 30 min, then in rabbit primary antibody rLr-PGRN-S1 (1:200) at 4 °C overnight. Then, the cells were incubated by Alexa Fluor 488-conjugated goat anti-rabbit IgG antibody (1:400). 4′,6-Diamidino-2-phenylindole (DAPI, 1:1000) was used for subsequent cell staining after washing using PBS three times. Finally, a drop of antifade solution was placed on the slides, and coverslips were mounted. Immunofluorescence was observed by using a Zeiss LSM 780 inverted microscope (Carl Zeiss, Inc).

### Fluorescence-activated cell sorting (FACS) analysis

Firstly, we used 90% methanol to fix the lamprey SB and liver cells at 25 °C for 20 min. Next, the cells were incubated in anti-rLr-PGRN-S1 (1:200) as primary antibody to incubate cells for 1 h, followed by fluorescein isothiocyanate (FITC)-conjugated donkey anti-rabbit IgG (1:500) as secondary antibody for 45 min at 25 °C in the dark. Finally, the cells were then rinsed three times with PBS and analyzed using a FACSAria flow cytometer (BD Biosciences). Gates were set using a control group. FlowJo analysis software (Tree Star) was used to analyze the data.

### Quantitative real-time PCR (qRT-PCR)

Tricaine methanesulfonate (0.05%; MS-222; 3-aminobenzoic acid ethyl ester, Sigma) was used to anesthetize immunized and normal lampreys, and tissues and cells were gathered. Lamprey epidermis was injured with a blade, and skin tissue samples were collected after 0, 1, and 2 days. The spinal cord was cut using a blade, and a spinal cord injury (SCI) sample was obtained from the back between the fourth and fifth gill holes of the lamprey. At 6 h, 1 day, and 3 days, 1-cm-thick tissue on the left and right sides of the wound was collected after anesthesia by using the above methods. Total RNA was obtained by using TRIzol, and reverse transcription was carried out as described earlier. The transcriptional levels of the PGRN family were detected base on the cDNA. PCR was performed with a TaKaRa SYBR PrimeScript RT-PCR kit. All experiments were carried out in triplicate. *L-gapdh* was used as internal control.

### Transwell migration, wound healing, and cell proliferation analysis

The chemotactic activity of rLr-PGRN-S1 on lamprey SB cells/human umbilical vein endothelial cells (HUVECs) was detected using transwell chambers (pore size 8 μm) without polyvinylpyrrolidone (Corning, NY, USA). Lamprey SB cells/HUVECs were layered in the upper chamber, and a solution containing PBS or rLr-PGRN-S1 was added to the lower chamber, followed by incubation for 24 h at 18 °C. A 4% paraformaldehyde solution was used to fix the invasive cells, and 0.1% crystal violet was used for staining. The cells were then analyzed using ImageJ software. The cell migration assay in each trial was conducted three times (with ten random fields per well). For the wound healing assay, HUVECs were seeded in a well dish. Line wounds were created across the cell layer in each well by using a 200-μL plastic tip. Next, serum-free Dulbecco’s modified Eagle’s medium (DMEM) was added, followed by treatment with rLr-PGRN-S1 or PBS. Cell motility was measured from photographs taken under a light microscope at 0, 1, and 2 days after wound making. HUVECs were cultured at density of 2 × 10^3^ per well on 96-well plates followed by addition of rLr-PGRN-S1. On days 0–2, the cell growth rate was determined using the cell counting kit-8 (CCK-8, B34304, Bimake) according to manufacturer’s instruction.

### siRNA delivery in lamprey embryos and spinal cord

Fertilized lamprey embryos were microinjected with *Lr-pgrn-s1* siRNA (Gene Pharma Co., Ltd., Shanghai, China). One- or two cell-stage eggs were microinjected with 10 nL of each siRNA at 40 mM concentration then kept at −80 °C until qRT-PCR. The knockdown efficiency of three types of siRNA was measured after extracting RNA from 30 gastrula embryos and quantifying mRNA levels using qRT-PCR. The siRNA with the highest knockdown efficiency was chosen for follow-up experiments. For siRNA delivery to the spinal cord, lampreys were anesthetized and placed on a clean laboratory table. An incision was then made between the third and fourth plug holes by using a sterilizing blade. We observed under a microscope whether the spinal cord was cut open at the fracture, and sutured using sterile needle and thread. Then the wound and swimming behaviors of lamprey were observed. According to Entranster TM-in Vivo’s instruction (Engreen Biosystem Co, Ltd.), the transfection reagent was mixed with siRNA. siRNA was injected at 2.7 μg/g concentration, after which paraffin sections and RNA samples were collected. The paraffin tissue was decolorized with gradient alcohol and embedded.

### RNA extraction, sequencing, annotation, and differential gene expression analysis

Total RNA was obtained from embryo cells by following the aforementioned method, and the RNA integrity number was tested using an Agilent Bioanalyzer 2100 (Agilent Technologies, USA). We then used the TruSeq stranded mRNA LT sample prep kit (Illumina, San Diego, CA, USA) to establish libraries based on the protocol. High-throughput transcriptome sequencing was performed by OE Biotech Co., Ltd. (Shanghai, China). The fragments per kilobase of transcript per million mapped reads (FPKM) of each gene was determined using Cufflinks, and the read counts of each gene were acquired by using HTSeq-count. The DESeq (2012) R package was used for differential expression analysis, with fold change > 2 and *P*-value < 0.05 set as the threshold. The expression patterns of differentially expressed genes (DEGs) from different groups were determined by hierarchical clustering analysis. Kyoto Encyclopedia of Genes and Genomes (KEGG) pathway and Gene Ontology (GO) enrichment analysis of DEGs were carried out using the R package according to a hypergeometric distribution.

### BIAcore analysis

A buffer (pH 4.0) was used to couple the rL-TNFR protein to the second channel of the CM5 chip, while the first channel was considered a reference channel. The analyzer rLr-PGRN-S1 protein was diluted by HBS-EP+ buffer, and the final rLr-PGRN-S1 protein solution concentration gradient was set to 162.5 nM, 325 nM, 650 nM, 1300 nM, and 2600 nM. The Biacore T200 (General Electric Company, USA) was used to analyze the data.

### Statistical analysis

All data are presented as mean ± standard error of the mean (SE) according to independent assays. Differences between treatment groups were analyzed by Student’s *t*-test. Differences were considered significant at *P* < 0.05 (*P < 0.05, **P < 0.01).

## Results

### Identification and sequence analysis of *pgrn* genes

**Fig. 1:**
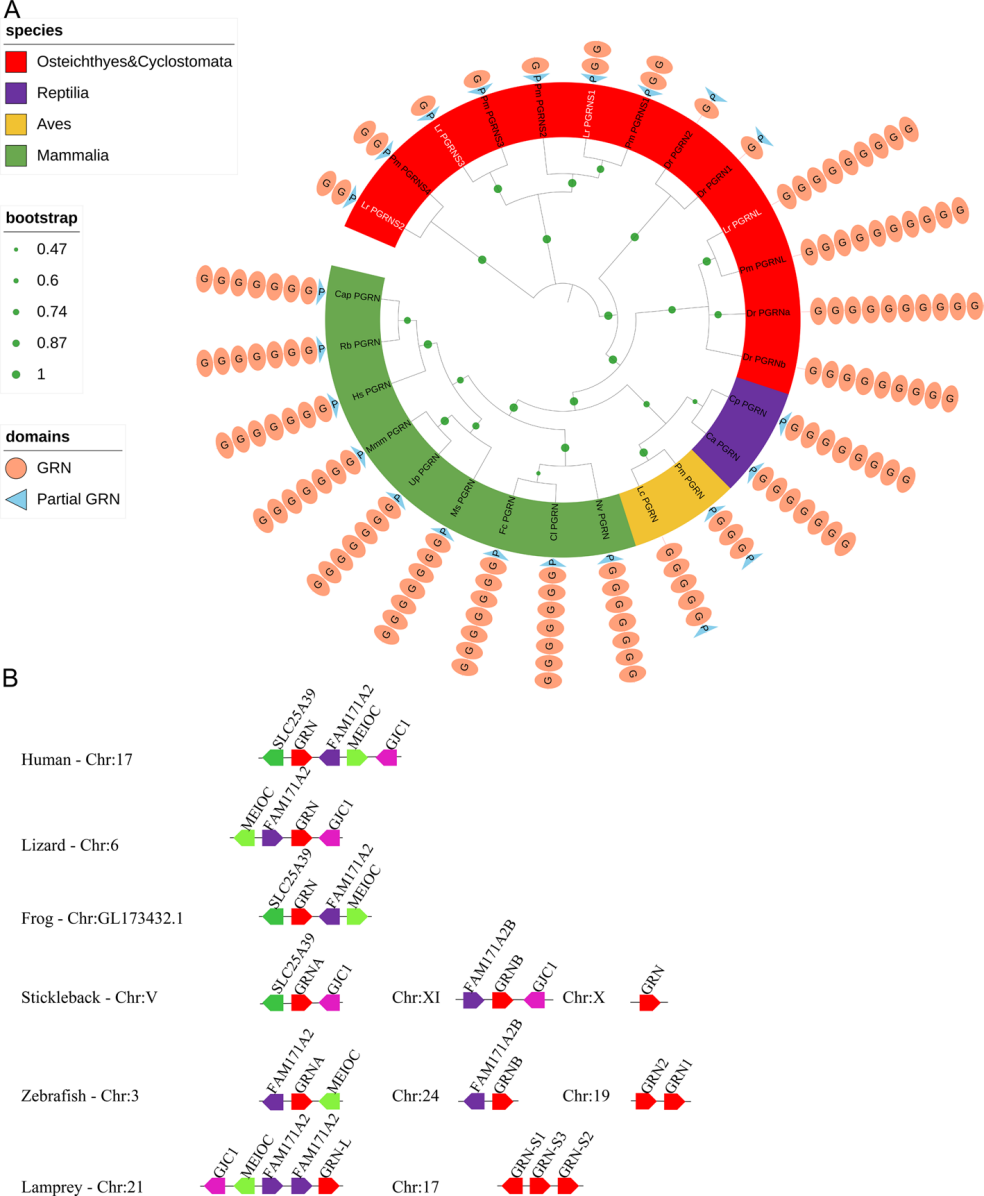
Phylogenetic tree and synteny analysis of the PGRN family. **A** Topological analysis of the resulting NJ tree with PGRNs of jawless and jawed vertebrates, including Mammalia (green background), Aves (yellow background), Reptilia (purple background), Osteichthyes, and Cyclostomata (red background). Light-salmon ellipse with G: domains of PGRN; light blue triangle with P: partial PGRN domain. The green circle on the branch points of the phylogenetic tree represents bootstrap values; the larger the circle area, the higher the bootstrap value. **B** Collinear analysis of *pgrn* genes in jawless and jawed vertebrates. The pentagon represents a gene, and identical genes are represented by the same color. The black line represents chromosome (Chr). The right side of the lamprey *pgrn-l* was considered to be the positive direction

To reveal the origin of granulin modules, and the features of early granulin-containing proteins, we screened for the vertebrate distribution and evolution of *pgrn* genes using the NCBI databases and our databases [29]. We identified only one *pgrn* gene in the mammalian genome. Unlike mammalian *pgrn*, we identified four *pgrn* genes in the genome of reissner lamprey (*Lethenteron reissneri*, Lr): one long-form *pgrn*, named *Lr-pgrn-l*, and three short-form *pgrns*, named *Lr-pgrn-s1–s3*. These genes were then cloned using the primers shown in Additional file 1: Table S1. Similarly, four paralogs, two long-form *pgrn* (*progranulins-A* and *-B*) and two short-form *pgrns* (*progranulins-1* and *-2*), were characterized in zebrafish [30]. Interestingly, four short-form *pgrns* (*Pm-pgrn-s1–s4*) were found in the sea lamprey (*Petromyrzon marinus*) [18], and *Pm-pgrn-s1* and *-s2* shared high sequence similarity (Additional file 1: Fig. S1). This was speculated to be caused by a mistake in sequencing or genome sequencing assembly. Both lamprey and teleost contained the maximum types of *pgrn* across all species [18]; this may be due to evolutionary adaptation. We conducted a phylogenetic analysis using the full-length sequences of these proteins. The sequences are presented in Table 1. The topology of the resulting NJ tree (Fig. 1A) suggests that the PGRNs of jawed and jawless vertebrates, containing Mammalia, Aves, Reptilia, Osteichthyes, and Cyclostomata, are unequivocally distributed into several groups following their evolutionary positions. Moreover, the lamprey short-form PGRNs were positioned in the outer group of the vertebrate PGRNs (Fig. 1A).

**Table 1:** Sequences of PGRNs used in phylogenetic tree analysis

Abbreviation	Full name	Accession number
Hs	*Homo sapiens*	NP_002078.1
Cap	*Colobus angolensis palliatus*	XP_011795487.1
Rb	*Rhinopithecus bieti*	XP_017731553.1
Ms	*Mus spretus*	BAE30030.1
Mmm	*Marmota marmota marmot*	XP_015351073.1
Nv	*Neovison vison*	XP_044103711.1
Up	*Urocitellus parryii*	XP_026268179.1
Fc	*Felis catus*	XP_011287550.2
Cl	*Canis lupus dingo*	XP_025295815.2
Lc	*Lepidothrix coronata*	XP_017694776.1
Pm	*Parus major*	XP_015506502.1
Ca	*Chelonoidis abingdonii*	XP_032651905.1
Cp	*Crocodylus porosus*	XP_019409476.1
Pf	*Poecilia formosa*	XP_007570945.1
Dr	*Danio rerio*	AAM00265.3
		XP_021325789.1
		NP_997921.1
		NP_001018638.2
Pm	*Petromyzon marinus*	XP_032824369.1
		XP_032806355.1
		XP_032806356.1
		XP_032806359.1
		XP_032806364.1

As shown in Fig. 1A, in contrast to mammalian PGRNs, the four reissner lamprey paralogs included successive 9, 2.5, 2.5, and 1.5 GRN modules in Lr-PGRN-L and Lr-PGRN-S1–S3, respectively. Human PGRN contains seven GRN modules in the order GRN (G-F-B-A-C-D-E), named HsGRN-1, 2, 3, 4, 5, 6, and 7 in this research. Vertebrate PGRN structure contains tandem module repetitions. To investigate pathways that might have resulted in their current modular architecture, the relationships among these modules were identified by NJ tree analyses according to the various granulin modules in vertebrates (Additional file 1: Fig. S2). Results of phylogenetic analysis demonstrated that modules 1 and 7 (GRNs G and E) were located in the outgroup, supporting the theory that the first and last modules (modules 1 and 7) are the most primitive. Furthermore, Lr-PGRN-S1-1 and Lr-PGRN-S2-1 were distributed in the GRNG cluster. To provide insights into the evolutionary landscape of the *pgrn* genes, the adjacent gene environment of lamprey and other vertebrate *pgrns* genes was compared. Lamprey *pgrn-l* was located close to the genes (*FAM171A2*, *MEIOC*, and *GJC1*) that formed an abundant block of conserved synteny with human chromosome 17 (Human-Chr: 17), revealing an orthologous relationship with human *pgrn* (Fig. 1B). Three *Lr-pgrn-s* genes occurred on lamprey Chr17, lying immediately adjacent to each other along the chromosome, indicating that multiple *Lr-pgrn-s* genes resulted from local tandem repetition. Results of the genomic synteny analysis showed contiguous alignment with Chr 19 of zebrafish, near the short-form *pgrn* genes (*Dr-pgrn1* and *Dr-pgrn2*) of zebrafish and other higher vertebrates, and the short-form *pgrn* ortholog was lost in the tetrapod lineage (Additional file 1: Fig. S3). In the reissner lamprey genome, however, a syntenic relationship with zebrafish short-form *pgrn* could not be found.

### Expression profiles of *Lr-pgrn* genes in lamprey and immune response to pathogenic challenges

**Fig. 2:**
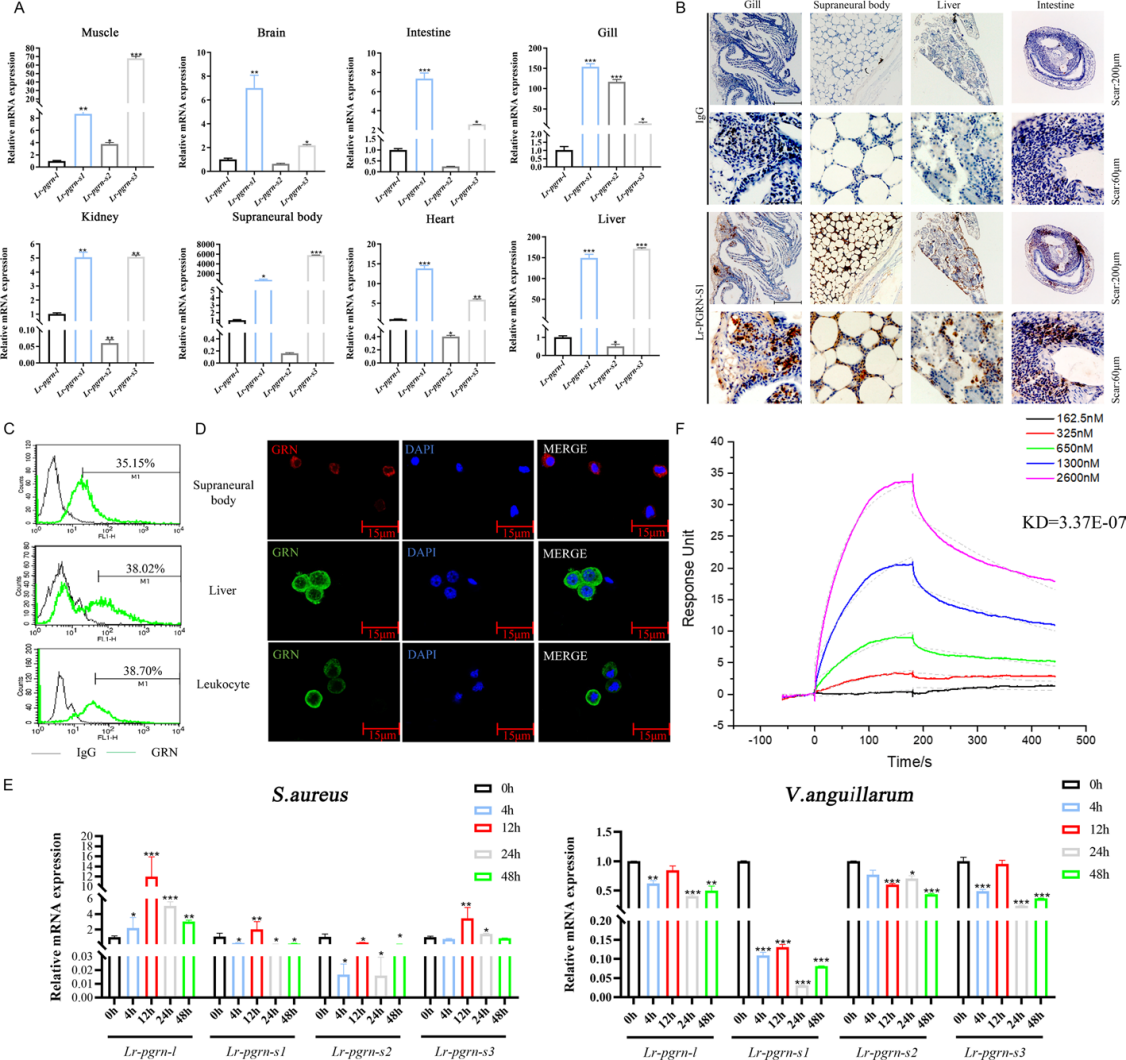
Expression of *Lr-pgrn* genes in normal lamprey tissue after pathogen stimulation. **A** The expression of *Lr-pgrn* genes in the brain, kidney, heart, and other lamprey tissues was measured with qRT-PCR. **B** The distribution of Lr-PGRN-S1 in the gill, supraneural body, kidney, and intestine tissues was measured by immunohistochemistry. The two expressions were observed in a ×10 and ×40 magnification field of view, respectively. Normal rabbit IgG was used as negative control. **C** Lr-PGRN-S1 protein expression in lamprey cells by FACS analysis. Representative contour plots were gated on Lr-PGRN-S1-positive cells (green line). The black line indicates cells with the IgG isotype control. **D** Immunofluorescence detection of the cellular location of Lr-PGRN-S1. Light blue: nucleus; light green and red: Lr-PGRN-S1. **E** The expression level of *Lr-pgrn* genes in lampreys after *S. aureus* and *V. anguillarum* stimulation during 0, 4, 12, 24, and 48 h as determined by qRT-PCR and normalized to *gapdh* expression. **F** Interaction of rLr-PGRN-S1 with rL-TNFR as determined by BIAcore analysis. The KD values were measured using the BIA evaluation version 3.0 software. The data are presented as mean ± standard deviation (SD) based on three independent samples with three replicates per sample. Probabilities of statistical differences between experimental groups in all bar graphs were determined by Student’s *t*-test. *P < 0.05, **P < 0.001, ***P < 0.0001

To verify the tissue-wise distribution of *Lr-pgrn-l* and *Lr-pgrn-s1–s3* in lampreys, we detected the transcriptional levels of these genes in normal tissues (Fig. 2A). The primers used are shown in Additional file 1: Table S1. We found that *Lr-pgrn-l* and *Lr-pgrn-s1–s3* showed different expression levels, with those of *Lr-pgrn-s1* and *Lr-pgrn-s3* being higher than those of *Lr-pgrn-l* and *Lr-pgrn-s2*. Additionally, *Lr-pgrn-s1* and *Lr-pgrn-s3* were mainly located in immune-related tissue cells, such as the supraneural body, leukocytes, and liver. Then, recombinant proteins were expressed, and their expression levels were determined using anti-Lr-PGRN-S1 polyclonal antibodies (Additional file 1: Figs. S4 and S5). Interestingly, Lr-PGRN-S1 was found to participate in modulating cell proliferation and migration (Fig. 4), so the tissue-wise distribution and cellular localization of Lr-PGRN-S1 were further investigated via immunohistochemistry, FACS, and confocal microscopy analysis using a specific anti-Lr-PGRN-S1 antibody. Immunohistochemical analysis showed that Lr-PGRN-S1 was localized in the gill, supraneural body, liver, and intestine tissues and was mainly expressed in the myeloid cells of the supraneural body, bases of the gill filaments, the gut groove tissues, and spaces of renal tubules in the kidneys (Fig. 2B). FACS analysis further confirmed that Lr-PGRN-S1 was expressed in the supraneural body, liver cells, and leukocytes (Fig. 2C), mainly in the cytoplasm, as determined by confocal microscopy (Fig. 2D).

The role of *Lr-pgrn* genes in the lamprey immune system remains unclear. To investigate the role of *Lr-pgrn* genes in the innate immune responses in lampreys, the expression levels of *Lr-pgrn* genes in response to experimental exposure to different pathogens were detected using qRT-PCR (Fig. 2E). The results revealed that the expressions of *Lr-pgrn-l*, *Lr-pgrn-s1*, and *Lr-pgrn-s3* mRNA were significantly upregulated at 12 h in the *S. aureus* immune group. Subsequently, the transcription levels of the genes were downregulated after 12 h. In the *V. anguillarum* immune group, the overall trend of the *Lr-pgrn-l*, *Lr-pgrn-s1*, *Lr-pgrn-s2*, and *Lr-pgrn-s3* transcription levels were reduced or normal. These results indicate that the lamprey *Lr-pgrn* genes might participate in the regulation of various signals by altering gene transcriptional levels in response to pathogen invasion. Previous studies have shown that PGRN has also emerged as a critical immune-regulatory molecule in regulating immune signaling pathways by binding the tumor necrosis factor receptor (TNFR) [31]. To investigate whether PGRN and TNFR interact in lamprey, we established the rL-TNFR protein [32] bound to an assay chip using surface plasmon resonance technology (Fig. 2F). rLr-PGRN-S1 at different concentrations served as the analyte, and analysis by affinity kinetics fitting was carried out by flowing rLr-PGRN-S1 protein along the rL-TNFR-bound chip. The affinity of KD was 3.37 × 10^−7^, indicating a strong interaction between Lr-PGRN-S1 and L-TNFR.

### RNA-seq for *Lr-pgrn-s1* target identification in embryo cells

**Fig. 3:**
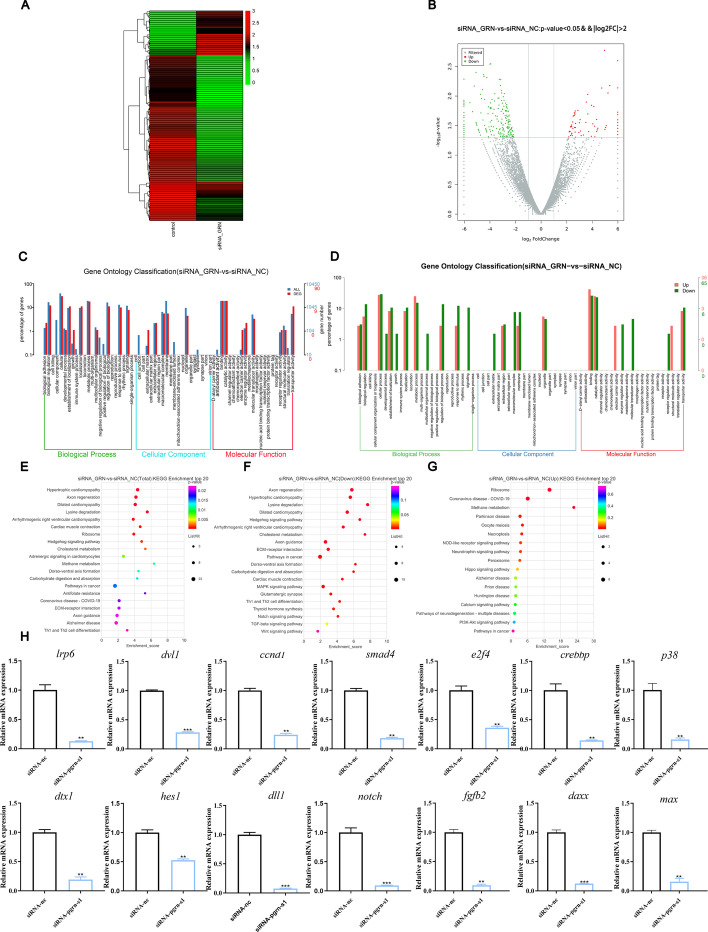
Transcriptome analysis and signaling pathway validation after *Lr-pgrn-s1* knockdown in embryonic cells. **A** Heatmap showing the expression of 100 genes in the control and experimental groups. Color intensity indicates expression levels. The color scale adjacent to the heatmap indicates the raw *Z*-score, ranging from green (low expression) to red (high expression). The similarity between individuals with hierarchical clustering is shown above the heatmap. **B** Volcano plot of differentially expressed genes (DEGs) between the experimental and control groups. DEGs with *p*-value < 0.05 and |log_2_ FC| > 2 were screened. One dot indicates one gene, red dots represent upregulated genes, green dots represent downregulated genes, and black dots indicate undifferentiated genes in the volcano. The smaller the *q-*value, the larger the −log(*q*-value), and the more significant the gene differentially expressed between the experimental and control groups. **C**, **D** GO analysis of the transcriptomic data of *Lr-pgrn-s1*knockdown of embryonic cells. The histogram presentation of GO classification indicates transcripts in numbers after being annotated in three groups: CC, MF, and BP. **E**–**G** KEGG analysis of upregulation and downregulation of TOP20 in the transcriptome of embryonic knockdown *Lr-pgrn-s1*. The top enrichment pathways are presented in the advanced bubble chart. The *X*-axis label represents the enrichment score, while the *Y*-axis label represents the pathway. The size of the bubble represents the number of genes enriched in the KEGG terms, while the color represents the FDR *P*-value of KEGG terms. **H** qRT-PCR analysis of genes related to the MAPK, Notch, Wnt, and TGF-β signaling pathways. Mean ± SD is shown (*n* = 3 per group). The probability of statistical differences between the experimental groups was determined using Student’s *t*-test. *P < 0.05, **P < 0.001, ***P < 0.0001

In a preliminary study, *Lr-pgrn-s1* showed higher mRNA expression levels than that of *Lr-pgrn-l*, *Lr-pgrn-s2*, and *Lr-pgrn-s3* in gastrula and cephalic phases during embryologic development, indicating that *Lr-pgrn-s1* might be involved in all phases of cell proliferation and differentiation (Additional file 1: Fig. S6A). Because of a lack of lamprey cell lines, to clarify the function of *Lr-pgrn-s1* in cell proliferation and the underlying molecular pathways, we conducted RNA sequencing analysis of *Lr-pgrn-s1* in lamprey embryo cells from the gastrula phase injected with siRNA (siRNA_GRN) and NC siRNA (siRNA_NC). The experiments were conducted in triplicates (ID: PRJNA803319). The primers used are shown in Additional file 1: Table S2. The mRNA expression levels of *Lr-pgrn-s1* in the cells decreased significantly after siRNA_GRN treatment compared with the control group (Additional file 1: Fig. S6B). The siRNA_GRN and siRNA_NC groups were resolved from each other in the cluster analysis (Fig. 3A). To investigate the DEGs between the siRNA_GRN and siRNA_NC groups, we applied the criteria of adjusted |log_2_ fold change| > 2 and *p* < 0.05. A total of 268 DEGs (58 upregulated and 210 downregulated) between the siRNA_GRN and siRNA_NC groups were identified (Fig. 3B). The DEGs were subjected to GO functional enrichment analysis. The top 20 GO terms of the cellular process, developmental process and metabolic process (biological process, BP), membrane and organelle (cellular component, CC), and binding, catalytic activity and transporter activity (molecular function, MF) are shown in Fig. 3C, D. Furthermore, KEGG enrichment analysis of three groups (GRN-vs-siRNA total, down, and up) (Fig. 3E–G) revealed four significant pathways associated with cell differentiation, including the Notch, MAPK, Wnt, and TGF-β significant pathways (Additional file 1: Fig. S7).We validated the RNA sequencing results by qRT-PCR of randomly selected downregulated genes, including *lrp6*, *dvl1*, *ccnd1*, *smad4*, *e2f4*, *crebbp*, *p38*, *dtx1*, *hes1*, *dll1*, *notch*, *fgfb2*, *daxx*, and *max* (Fig. 3H).The primers used are shown in Additional file 1: Table S1.

### Lr-PGRN-S1 promotes cell proliferation and migration, involved in wound repair

**Fig. 4:**
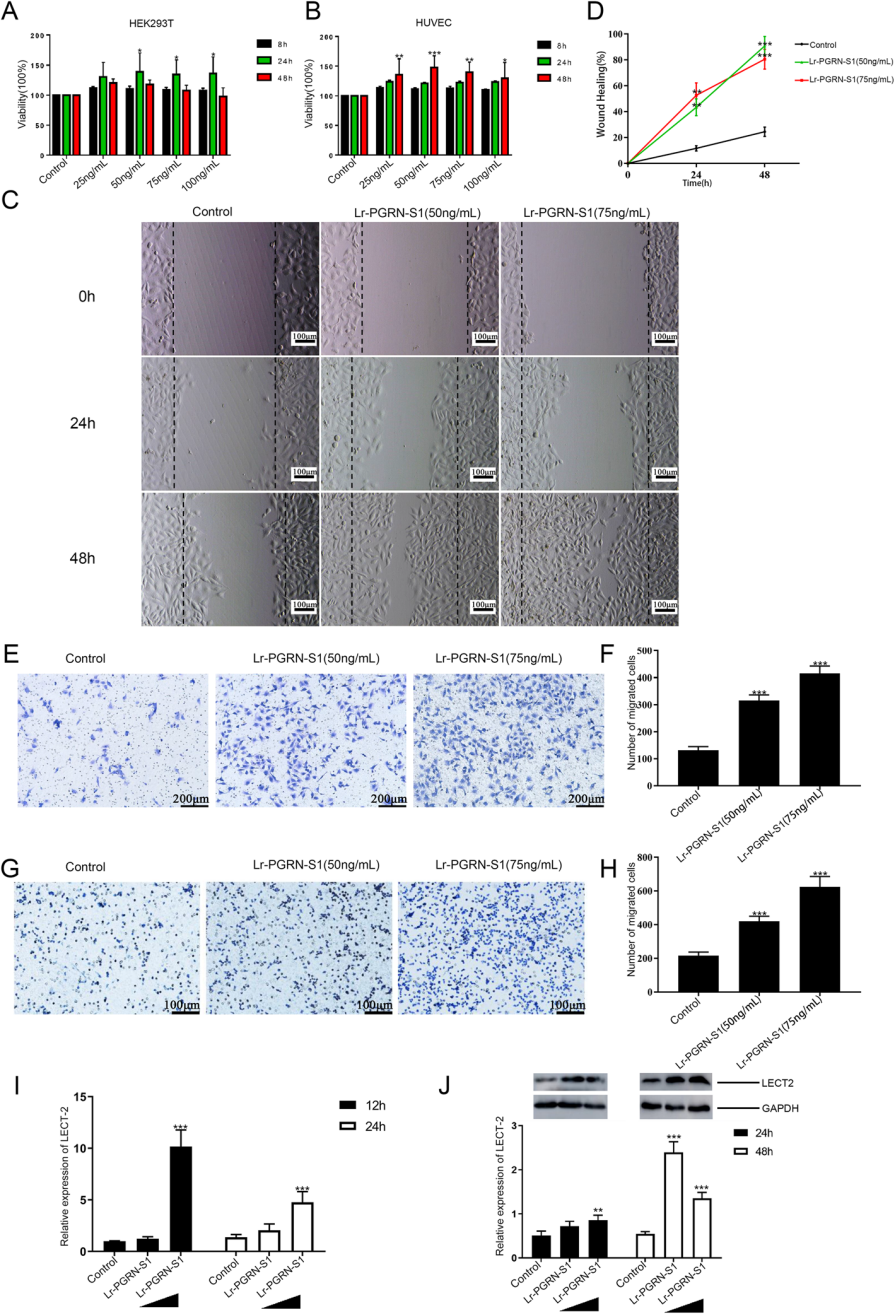
Lr-PGRN-S1 promotes cell proliferation and migration. **A**, **B** rL-PGRN-S1 promoted HEK293T cell and HUVEC proliferation. HEK293T cells and HUVECs were treated with rLr-PGRN-S1 of different concentrations. The viability of the cells was detected using the CCK-8 assay. **C** Wounds were produced by scratching the bottom of the cell culture plate using a pipette tip. Cells were incubated with IPC for up to 48 h. To observe the wound healing effect, the area around the wounds was assessed at indicated time points. PBS was used as control. **D** Line chart showing the statistics of the results in C. **E** Transwell assay showing promotion of migration in rL-PGRN-S1-treated HUVECs; **F** graph of rL-PGRN-S1 promotion of HUVEC migration. **G** Transwell assay showing promotion of migration in rL-PGRN-S1-treated lamprey supraneural body. **H** Statistical graph of rL-PGRN-S1. **I** Effect of rL-PGRN-S1 on expression of chemokine LECT2 as determined by qRT-PCR. **J** Effect of rL-PGRN-S1 on expression of chemokine LECT2 as determined by western blot. The lower histogram shows the statistics of western blot. The probability of statistical differences between experimental groups was determined by Student’s *t*-test. All data presented as mean ± SD based on three independent samples with three replicates per sample. *P < 0.05, **P < 0.001, ***P < 0.0001

**Fig. 5:**
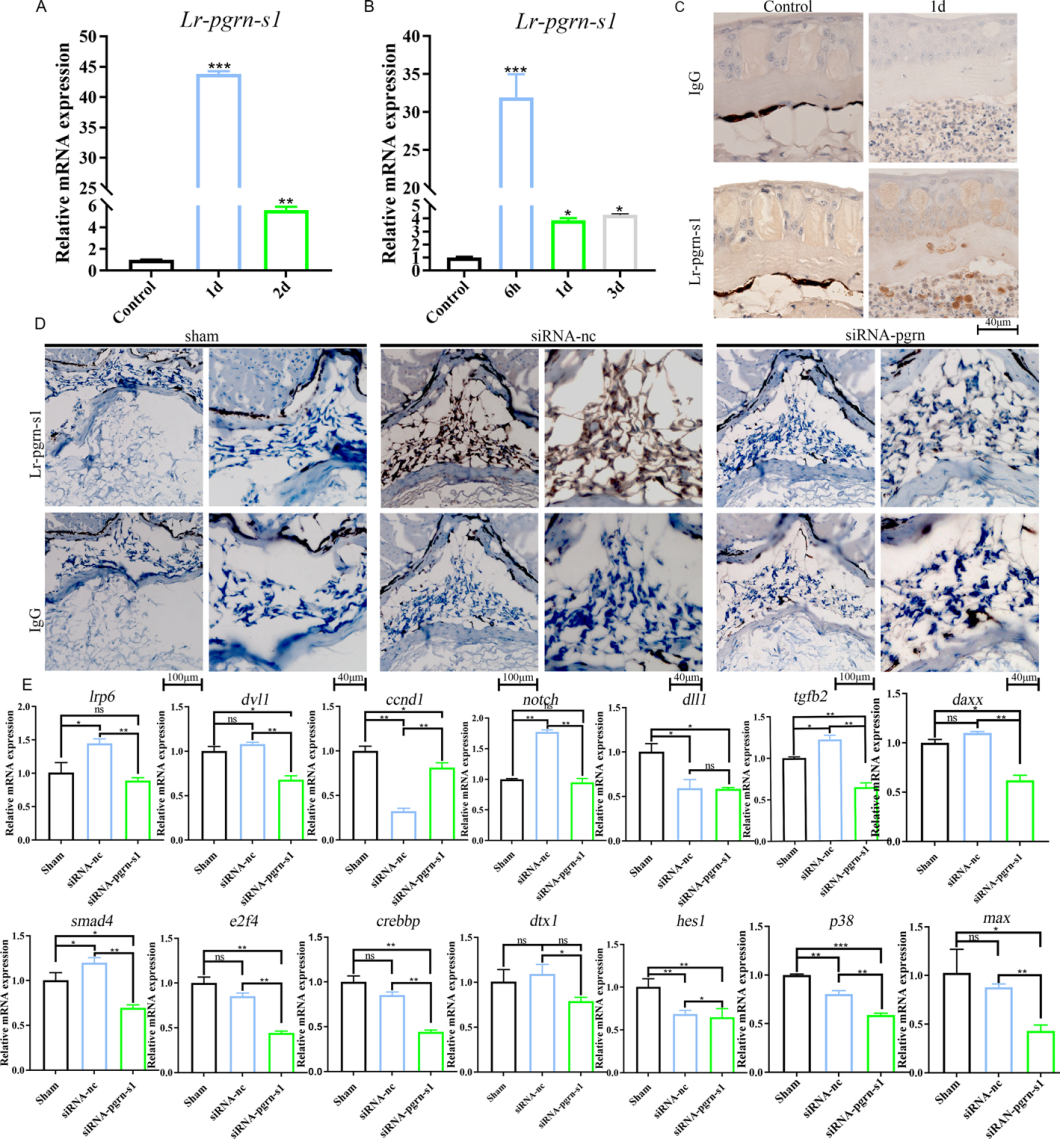
Expression of *Lr-pgrn-s1* after lamprey skin and spinal cord injury. **A** Expression of *Lr-pgrn-s1* in lamprey skin lesions 1 and 2 days after injury. **B** Expression of *Lr-pgrn-s1* in lamprey 6 h, 1 day, and 3 days after spinal cord injury by qRT-PCR. **C** Tissue-wise localization of Lr-PGRN-S1 1d after skin injury by immunohistochemistry. Pictures taken at ×40 magnification. **D** Immunohistochemistry analysis shows *Lr-pgrn-s1* was knocked down 6 h after lamprey spinal cord injury. Results under ×20 and ×40 microscopy in sham, NC (normal control), and Lr-PGRN-S1 groups. **E** qRT-PCR analysis of genes in MAPK, Notch, Wnt, and TGF-β signaling pathways. Statistical differences between experimental groups were detected by Student’s *t* test. All data presented as mean ± SD based on three independent samples with three replicates per sample. *ns* nonsignificant, *P < 0.05, **P < 0.001, ***P < 0.0001

Transcriptome sequencing was performed on HEK293T cells treated with rLr-PGRN-S1 to further analyze the mechanism underlying the Lr-PGRN-mediated cell proliferation and migration (ID: PRJNA803267). Lr-PGRN-S1 was involved in multiple significant migration-associated pathways, including the MAPK and PI3K-Akt signaling pathways, and leukocyte transendothelial migration, etc., in agreement with the result obtained above for the transcriptome sequencing (Additional file 1: Fig. S8). To investigate whether Lr-PGRN-S1 plays a role in cell proliferation, we used purified Lr-PGRN-S1 to treat HEK293T cells and HUVECs. Lr-PGRN-S1 stimulated significant proliferation of HEK293T cells at 24 h and HUVECs at 48 h (Fig. 4A and B, respectively). We further investigated the roles of Lr-PGRN-S1 in the migration of HEK293T cells with a “wound healing” motility assay in vitro. HEK293T cells were cultured in a serum-containing medium and wounded at 0 h. The cells were incubated with Lr-PGRN-S1 (50 ng/mL and 75 ng/mL) for 24 h and 48 h. We found that, compared with control group, Lr-PGRN-S1 induced cell migration in rLr-PGRN-S1-treated cells (Fig. 4C); the statistics of the “wound healing” motility assay are shown by a line graph (Fig. 4D). The transwell analysis suggested that HUVEC treated with Lr-PGRN-S1 possessed greater ability than the control group to pass through Matrigel-coated filters (Fig. 4E, F). This is consistent with the results obtained for lamprey supraneural body cells (Fig. 4G, H). Moreover, to shed light on the cell migration activity of Lr-PGRN-S1, the mRNA and protein expression levels of identified leukocyte cell-derived chemotaxin 2 (LECT2) [33] in lamprey supraneural body cells stimulated with Lr-PGRN-S1 were determined (Fig. 4I, J). We found that Lr-PGRN-S1-treated cells showed upregulated LECT expression. To investigate whether the expression of *Lr-pgrn-s1* is upregulated in response to wounding, we repeatedly detected *Lr-pgrn-s1* mRNA level by qRT-PCR and Lr-PGRN-S1 level by immunohistochemistry in the wound tissues of lamprey at several time points after skin and spinal injury. *Lr-pgrn-s1* mRNA expressions increased dramatically at 1 day (Fig. 5A) and 6 h (Fig. 5B) after injury, respectively, indicating the role of *Lr-pgrn-s1* in wound response. We also performed immunohistochemistry to identify the cell types showing upregulated Lr-PGRN-S1 expression on 1 day after skin wounding and found Lr-PGRN-S1 expression to be high in the fat layer, implying that Lr-PGRN-S1 might perform a function at early stages in the wound response (Fig. 5C). Lr-PGRN-S1 protein expression was also upregulated after spinal injury but was subsequently suppressed by siRNA (Fig. 5D). In addition, the mRNA expressions of genes associated with cell differentiation, including *lrp6*, *dvl1*, *ccnd1*, *smad4*, *e2f4*, *crebbp*, *p38*, *dtx1*, *hes1*, *dll1*, *notch*, *fgfb2*, *daxx*, and *max*, were downregulated by siRNA (Fig. 5E).

## Discussion

The *pgrn* genes are distributed across metazoans from sponges and cnidaria to mammals. However, they are absent from the genomes of some species such as the honey bee and fruit fly, the reasons for which remain unclear [18]. The human *pgrn* were first isolated from bone marrow extracts [34]. The main difference between lamprey *pgrn* and human *pgrn* is that, compared with human *pgrn*, lamprey *pgrn* encode for much shorter PGRN proteins, similar to the teleost. More paralogs in teleost fish have been extensively identified, supporting the theory of an extra duplication in the ray-finned lineage genome [35, 36]. Synteny block analysis has proved that zebrafish gene copies are usually located on diverse chromosomes that share conserved synteny blocks with those in the mammalian genome [18, 37]. In zebrafish, there are two precursors (PGRN A and PGRN B) carrying ten and nine GRN domains, respectively, which harbor a structure similar to human PGRN [30]. Hence, zebrafish PGRN A and B were considered to share a co-orthologous relationship with mammalian PGRNs. The *PGRN A* and *PGRN B* genes are believed to be derived from the duplication of large-scale chromosomal regions, which also resulted in several paralogous pairs in other fish species such as *Oryzias latipes* (medaka), *Xiphophorus maculatus* (southern platyfish), *Tetradon nigroviridis* (globe fish), etc. However, a single PGRN, that is Lr-PGRN-L in *Lethenteron reissneri* (reissner lamprey), is consistent with that found in mammals.

In addition, two shorter zebrafish *pgrn* genes, containing one full and one half GRN domains, had also been identified; however, these have been derived from genome duplication, positioned in tandem on the same chromosome, and are located near a *dlx* and *Hox* cluster gene, indicating that they originated from duplication of a chromosome harboring the *Hox* genes in an ancient organism [30]. Furthermore, several short-form *pgrn* genes in a genome are usually located in tandem on the same chromosome, suggesting that the *Lr-pgrn-s1–s3* genes originated through continuous duplication. As shown in Additional file 1: Fig. S3, there are conservative genomic blocks near the short-form *pgrn* in *Danio rerio* (zebrafish, *Dr-pgrn1* and *Dr-pgrn2*) and *Latimeria chalumnae* (coelacanth, *Lc-pgrn*). Thus short-form *pgrn* genes in zebrafish and coelacanth likely derive from a common ancestral gene. Nevertheless, the syntenic relationship was not identified in lamprey and is attributable to chromosomal rearrangement. The genomic sequence analysis of zebrafish and coelacanth [38] proved that various genes, which have functions specific to fish, were lost in tetrapod organisms. *pgrn* belong to a family of growth factors identified to mediate diverse biological processes. They are extremely conserved across the metazoans. Although large amounts of active research on PGRNs has focused primarily on mammals, they are highly conservative across metazoans [39]. The comparative analysis of PGRN activations revealed that a number of the activations identified in mammalian model systems might be similar to those of other species [19, 30]. Moreover, PGRN-like genes have been found in some major organisms, containing evolutionarily key organisms (e.g., slime molds and choanoflagellates), which form crucial nodes in the phylogeny of metazoans. In mammals, infected cells can secrete inflammatory cytokines that mediate the inflammatory signaling pathway. TNF-α is crucial to the inflammation pathway and plays diverse roles in the immune response [40]. PGRNs can regulate the TNF-α signaling pathway by combining with the cysteine-rich domains (CRDs) of the TNF receptor [41]. Furthermore, PGRNs regulate the inflammatory signal pathway of nuclear factor of kappa (NF-κB) by mediating protein kinase 1 and 2 (ERK1/2) [42]. We demonstrated that the lamprey *pgrn-s1* gene is involved in inflammatory response. Besides, Lr-PGRN-S1 can bind to L-TNFR with high affinity, which is pivotal for the effect of PGRNs.

Remarkably, in mammals, the expression of PGRNs is significantly increased in cases of wound and inflammation [10, 15], and damage repair is mediated by diversified regulatory growth factors and cytokines, which serve in the healing of skin or tissue wounds via phosphoinositide 3-kinases (PI3K) and mitogen-activated protein kinase (MAPK) activation and further induce the activation of genes related to cellular differentiation and proliferation [43–45]. *pgrn* expression is upregulate in injured skin tissues and maintained for a prolonged period [10, 15, 46]. In comparison, the expression level of *pgrn* is low in undamaged tissue but can be dramatically induced after injury. PGRN in skin wounds can prolong inflammatory infiltration, which can lead to aggregation of fibroblasts and blood vessels in wounds due to secretion of hepatocyte growth factor (HGF), TNF, and transforming growth factor-β (TGF-β). SCI entails two steps: initial damage such as mechanical shearing and extension of blood vessels and axons, followed by a cascade of secondary processes that results in the activation of glial cells, progressive lesion expansion, and infiltration of leukocytes, exacerbating tissue wound through the secretion of reactive oxygen species (ROS) and proinflammatory cytokines [47, 48]. While the initial injury is quick with no reversal, the secondary injury is adjusted through inflammatory effectors with time. Naphade et al. showed that PGRN was remarkably activated following SCI, primarily sourced from active microglia and macrophages after injury, and might be an alternative molecule for regulating secondary injury as well as subsequent repair of the damaged spinal cord [49]. Moreover, PGRN is also considered to be a potential therapeutic target in SCI [50]. In this research, we found that Lr-PGRN-S1 expression greatly increased in response to induced skin and spinal injury in lampreys, and Lr-PGRN-S1 could bind L-TNFR with high affinity, suggesting that it might be involved in the regulation of Lr-PGRN-S1 for damage repair. The results demonstrate that Lr-PGRN-S1 is involved in damage repair after skin and spinal cord injury in proper conditions. Understanding the precise molecular mechanism and underlying effects of PGRNs in skin and spinal cord injury repair warrants further research.

In summary, we unveiled the molecular evolution and tissue-wise distribution of lamprey PGRNs. Our results reveal that lamprey PGRN may be involved in the inflammatory response and play a role in damage repair. This study not only improves our understanding of the phylogeny of the vertebrate PGRN family but also provides new insight into the diverse functions of PGRN.

## Supplementary Information

**Additional file 1: Fig S1.** Amino acid and nucleotide sequence alignment of Pm-PGRN-S1 and Pm-PGRN-S2. A: Amino acid sequence alignment. B: Nucleotide sequence alignment. **Fig S2.** Phylogenetic tree analysis of PGRN domains in jawless and jawed mammals. Hs-PGRN is represented by the red background. Lr-PGRNs are represented by the blue background. Pm-PGRNs are represented by wathet-blue background. GRN A module: pink semiarc; GRN B module: atrovirens semiarc; GRN C module: blue semiarc; GRN D module: purple semiarc; GRN E module: light-red semiarc; GRN F module: wathet-blue semiarc; GRN G module: bottle-green semiarc. **Fig S3.** Collinear analysis of lamprey *pgrns1-s3*. The right side of the lamprey *pgrn-s2* is the positive direction. Identical genes are represented by the same color. A semicircle–rectangle represents a gene, and identical genes are represented by the same color. The black line represents chromosome (Chr). **Fig S4.** Expression and purification of rL-PGRN-S1 protein. A: rL-PGRN-S1 protein expression results. M: protein marker; 1: uninduced expression of *E. coli*; 2–7: induced expression of *E. coli*. B: rL-PGRN-S1 protein purification results. M: protein marker; 1: uninduced expression of *E. coli*; 2: induced expression of *E. coli*; 3: supernatant; 4–5: inclusion body; 6: filtered; 7: binding elution; 8: elution with 400 mmoL/L imidazole; 9: 0.2 μg/μL BSA. **Fig S5.** Titer detection of Lr-PGRN-S1 polyclonal antibody using ELISA. Serially diluted (1:20,000–1:640,000) polyclonal antibodies were tested against Lr-PGRN-S1 recombinant protein by ELISA. Pre-immune IgG was used as negative control (*n* = 3). Error bars indicate standard error of the mean (s.e.m.). **Fig S6.** Expression and knockdown efficiency of *Lr-pgrn* genes during embryonic development. A: Expression of *Lr-pgrn* genes in gastrula and cephalic stages detected by qPCR. B: Knockdown efficiency of *Lr-pgrn-s1* detected by qPCR. The probability of statistical differences between experimental groups was determined by Student’s *t*-test. Mean ± SD is shown (*n* = 3 per group). ns: nonsignificant, *P < 0.05, **P < 0.001, ***P < 0.0001. **Fig S7.** Molecular changes in the MAPK, Notch, Wnt, and TGF-β signaling pathways in KEGG enrichment analysis of transcriptomic data. Green represents downregulated genes; red represents downregulated genes. **Fig S8.** A: GO annotation map of DEG. Red represents biological processes; Green represents cellular components; Blue represents molecular functions. B: KEGG classification map of differential gene. Cellular processes: yellow; environmental information processing: purple; genetic information processing: pink; human diseases: red; metabolism: green; organismal systems: blue. **Table S1.** Primers used for PCR in this study. **Table S2.** Primers used for siRNA and vector linking in this study.

## Data Availability

The data used to support the findings of this research are included within the article. The data and materials in the current study are available from the corresponding author on reasonable request.

## Abbreviations

qPCR: Real-time quantitative polymerase chain reaction
siRNA: Small interfering RNA
RNA-Seq: RNA sequencing
DEG: Differentially expressed gene
BLASTX: Basic Local Alignment Search Tool X
FACS: Fluorescence-activated cell sorting
MF: Molecular function
CC: Cellular component
GO: Gene ontology
BP: Biological process
KEGG: Kyoto Encyclopedia of Genes and Genomes
ORF: Open reading frame
PVDF: Polyvinylidene difluoride
DEPC: Diethylpyrocarbonate
