# Correction: Granulin as an important immune molecule involved in lamprey tissue repair and regeneration by promoting cell proliferation and migration

**DOI:** 10.1186/s11658-022-00396-8

**Published:** 2022-10-31

**Authors:** Ruixiang Sun, Dong Wang, Yuxuan Song, Qingwei Li, Peng Su, Yue Pang

**Affiliations:** 1 College of Life Sciences, Liaoning Normal University, Dalian, 116081 China; 2 Lamprey Research Center, Liaoning Normal University, Dalian, 116081 China; 3 Collaborative Innovation Center of Seafood Deep Processing, Dalian Polytechnic University, Dalian, 116034 China

## Correction: Cellular & Molecular Biology Letters (2022) 27:64 https://doi.org/10.1186/s11658-022-00360-6

Following publication of the original article [1], the authors identified an error in Fig. 5. They reused the picture of NC group of Fig. 5D (published on line) in the PGRN-S1 group. Thus, they used the correct picture to replace it. The incorrect and the correct figure is given below.

The incorrect Fig. 5 is:
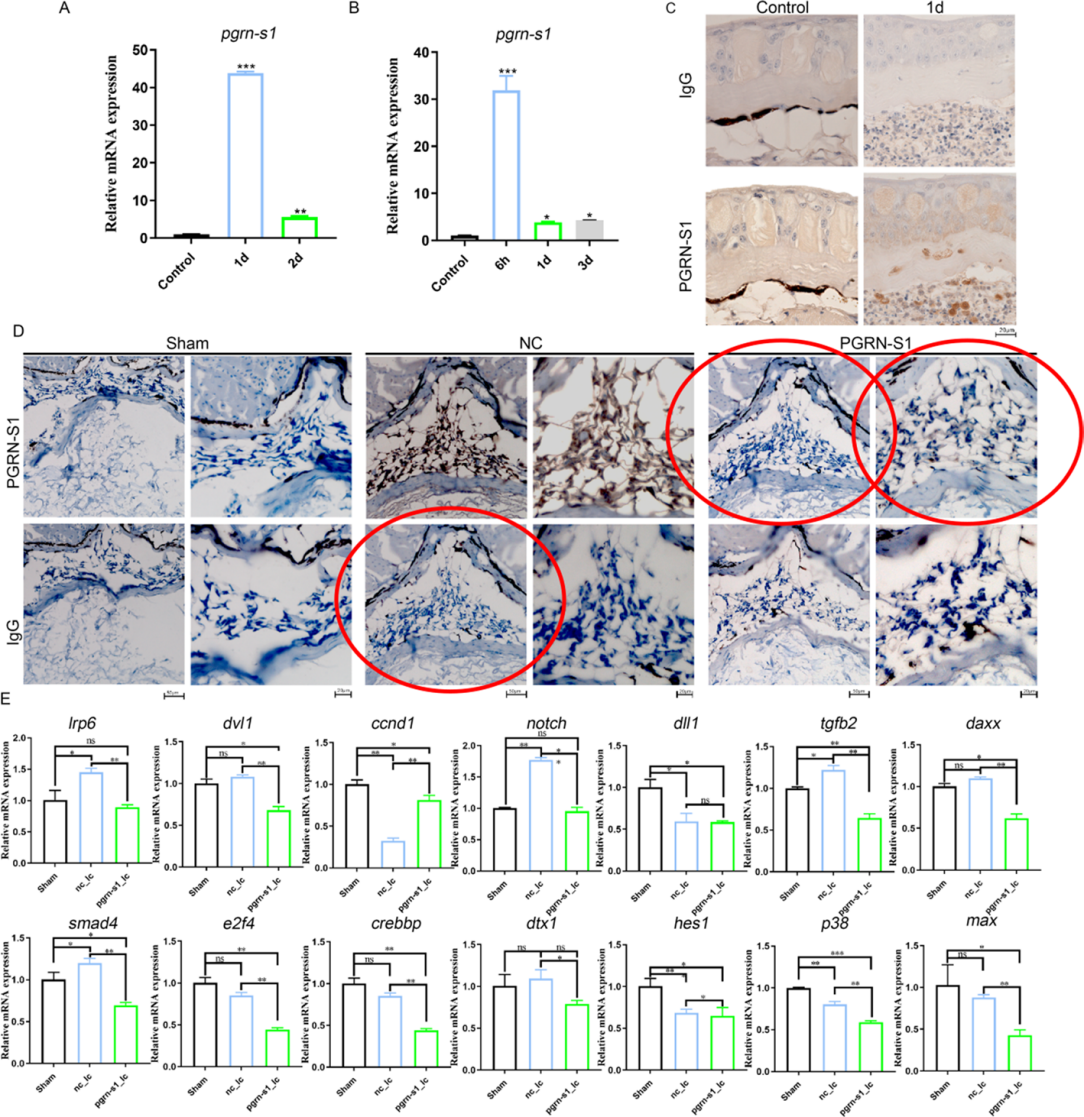


**Fig. 5** Expression of Lr-pgrn-s1 after lamprey skin and spinal cord injury. **A** Expression of Lr-pgrn-s1 in lamprey skin lesions 1d and 2d. **B** Expression of Lr-pgrn-s1 in lamprey spinal cord injury at 6 h, 1d and 3d by qRT-PCR. **C** Detection of tissue localization of Lr-PGRN-S1 in skin injury 1d by immunohistochemistry. Take pictures at 40× magnification. **D** Immunohistochemistry of Lr-pgrn-s1 was knocked down 6 h after lamprey spinal cord injury. Results under 20× and 40× microscopy in Sham, NC (normal control) and Lr-PGRN-S1 groups. **E** The qRT-PCR analysis of genes in MAPK, Notch, Wnt and TGF-β signaling pathways. The statistical differences between experimental groups were detected by the Student’s *t* test. All data were presented as the means ± SDs based on three independent samples with three replicates per sample. *ns* non-significant, *P < 0.05, **P < 0.001, ***P < 0.0001.

The correct Fig. 5 is:

**Fig. 5:**
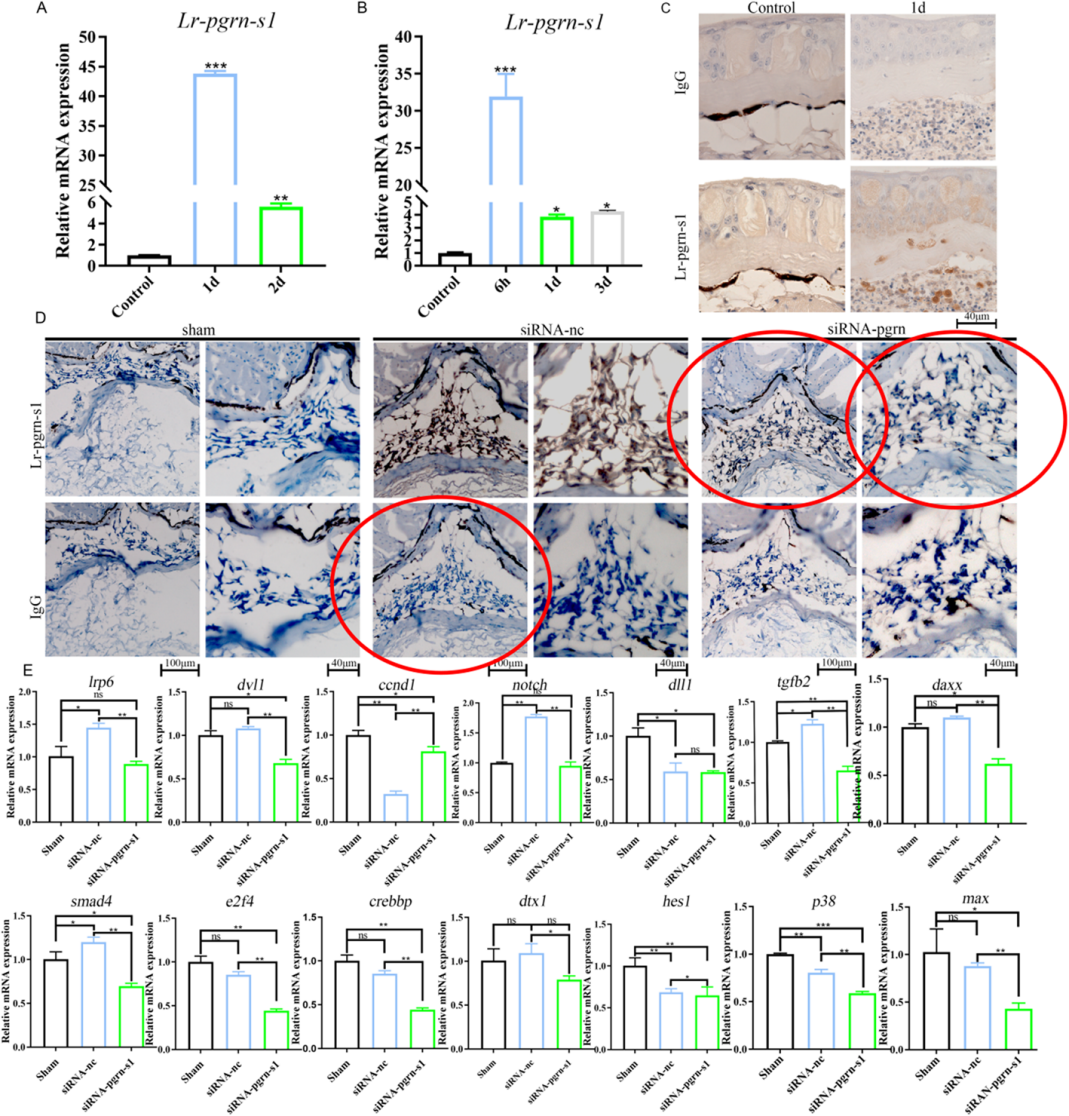
Expression of Lr-pgrn-s1 after lamprey skin and spinal cord injury. **A** Expression of Lr-pgrn-s1 in lamprey skin lesions 1d and 2d. **B** Expression of Lr-pgrn-s1 in lamprey spinal cord injury at 6 h, 1d and 3d by qRT-PCR. **C** Detection of tissue localization of Lr-PGRN-S1 in skin injury 1d by immunohistochemistry. Take pictures at 40× magnification. **D** Immunohistochemistry of Lr-pgrn-s1 was knocked down 6 h after lamprey spinal cord injury. Results under 20× and 40× microscopy in Sham, NC (normal control) and Lr-PGRN-S1 groups. **E** The qRT-PCR analysis of genes in MAPK, Notch, Wnt and TGF-β signaling pathways. The statistical differences between experimental groups were detected by the Student’s *t* test. All data were presented as the means ± SDs based on three independent samples with three replicates per sample. ns: non-significant, *P < 0.05, **P < 0.001, ***P < 0.0001
