# Supplementary material for: Granulin as an important immune molecule involved in lamprey tissue repair and regeneration by promoting cell proliferation and migration
Source: Cell Mol Biol Lett. 2022 Jul 30;27:64. doi: 10.1186/s11658-022-00360-6 (PMC9338584; doi:10.1186/s11658-022-00360-6)
Supplement: Supplementary file 1 — Additional file 1: Fig S1. Amino acid and nucleotide sequence alignment of Pm-PGRN-S1 and Pm-PGRN-S2. A: Amino acid sequence alignment. B: Nucleotide sequence alignment. Fig S2. Phylogenetic tree analysis of PGRN domains in jawless and jawed mammals. Hs-PGRN is represented by the red background. Lr-PGRNs are represented by the blue background. Pm-PGRNs are represented by wathet-blue background. GRN A module: pink semiarc; GRN B module: atrovirens semiarc; GRN C module: blue semiarc; GRN D module: purple semiarc; GRN E module: light-red semiarc; GRN F module: wathet-blue semiarc; GRN G module: bottle-green semiarc. Fig S3. Collinear analysis of lamprey pgrns1-s3. The right side of the lamprey pgrn-s2 is the positive direction. Identical genes are represented by the same color. A semicircle–rectangle represents a gene, and identical genes are represented by the same color. The black line represents chromosome (Chr). Fig S4. Expression and purification of rL-PGRN-S1 protein. A: rL-PGRN-S1 protein expression results. M: protein marker; 1: uninduced expression of E. coli; 2–7: induced expression of E. coli. B: rL-PGRN-S1 protein purification results. M: protein marker; 1: uninduced expression of E. coli; 2: induced expression of E. coli; 3: supernatant; 4–5: inclusion body; 6: filtered; 7: binding elution; 8: elution with 400 mmoL/L imidazole; 9: 0.2 μg/μL BSA. Fig S5. Titer detection of Lr-PGRN-S1 polyclonal antibody using ELISA. Serially diluted (1:20,000–1:640,000) polyclonal antibodies were tested against Lr-PGRN-S1 recombinant protein by ELISA. Pre-immune IgG was used as negative control (n = 3). Error bars indicate standard error of the mean (s.e.m.). Fig S6. Expression and knockdown efficiency of Lr-pgrn genes during embryonic development. A: Expression of Lr-pgrn genes in gastrula and cephalic stages detected by qPCR. B: Knockdown efficiency of Lr-pgrn-s1 detected by qPCR. The probability of statistical differences between experimental groups was determined by St [file 11658_2022_360_MOESM1_ESM.docx]

**Additional figures**


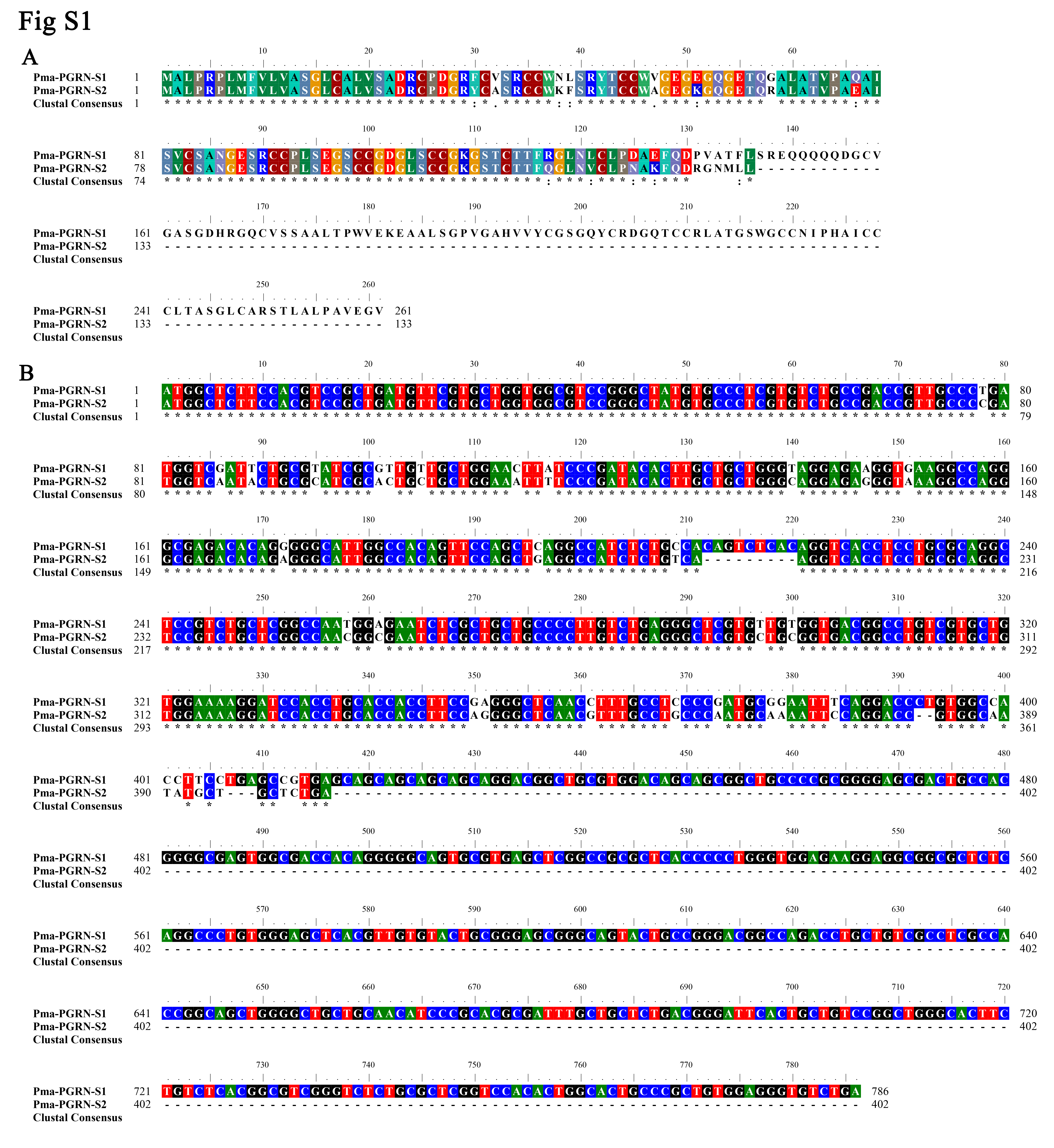


**
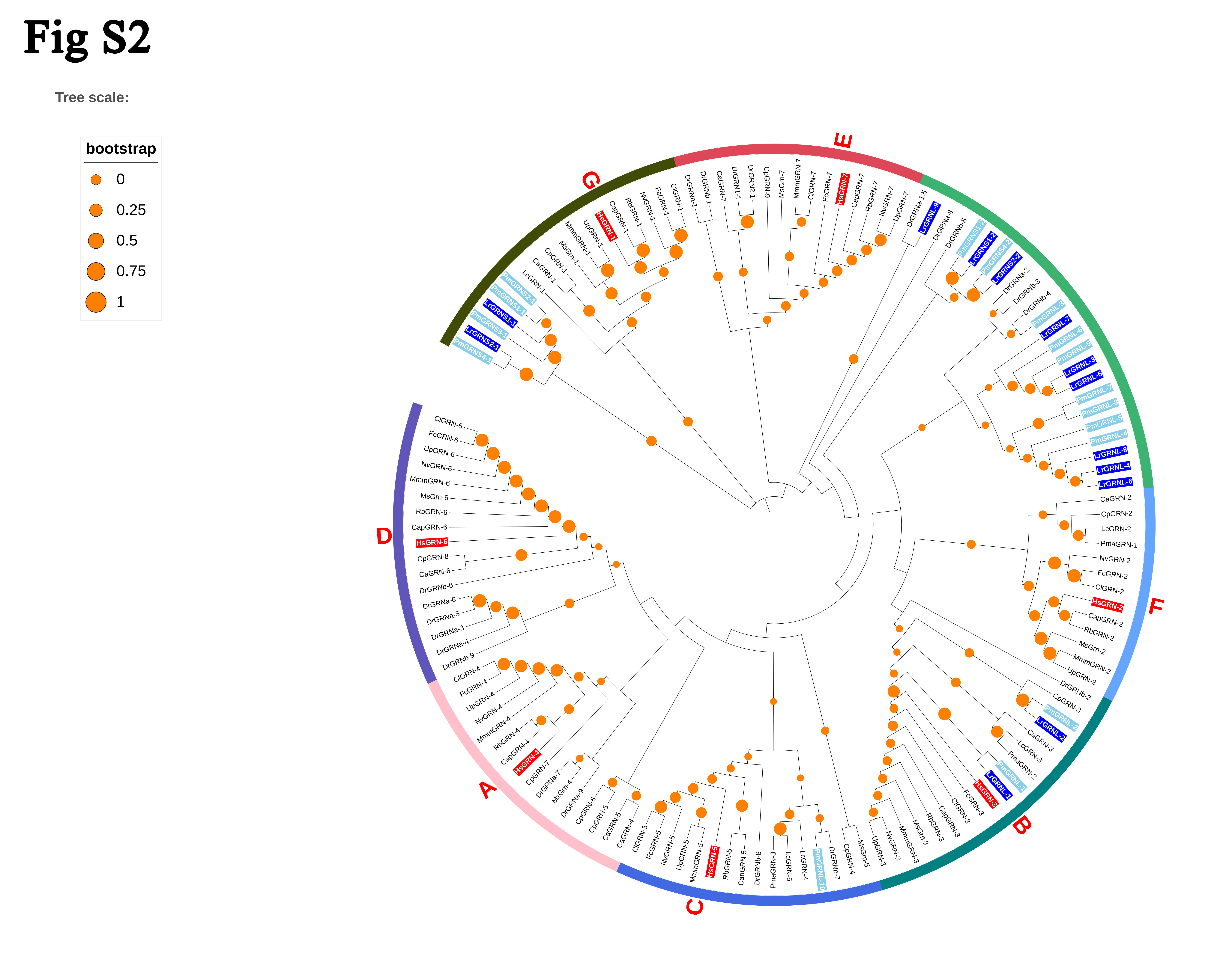
**

**
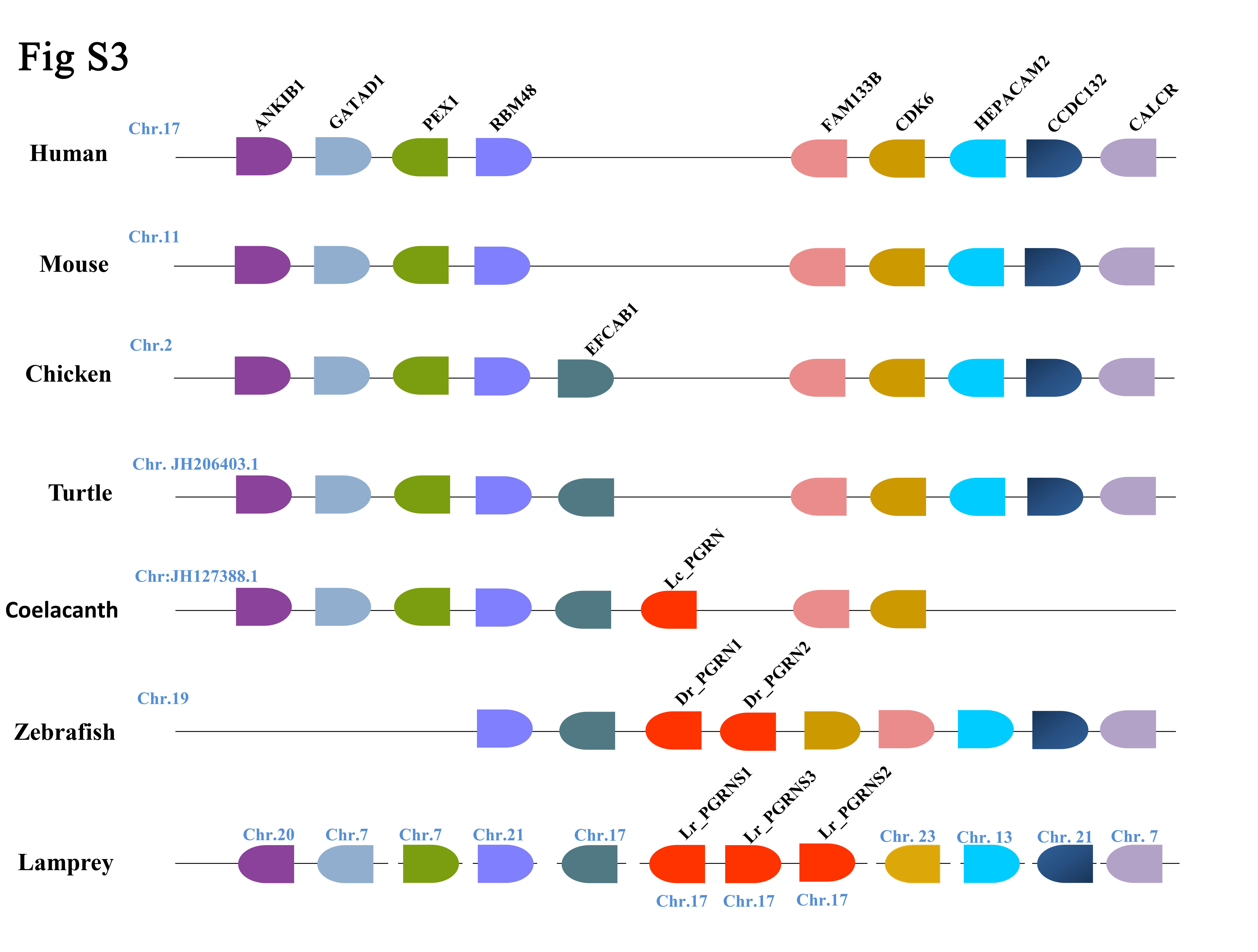
**

**
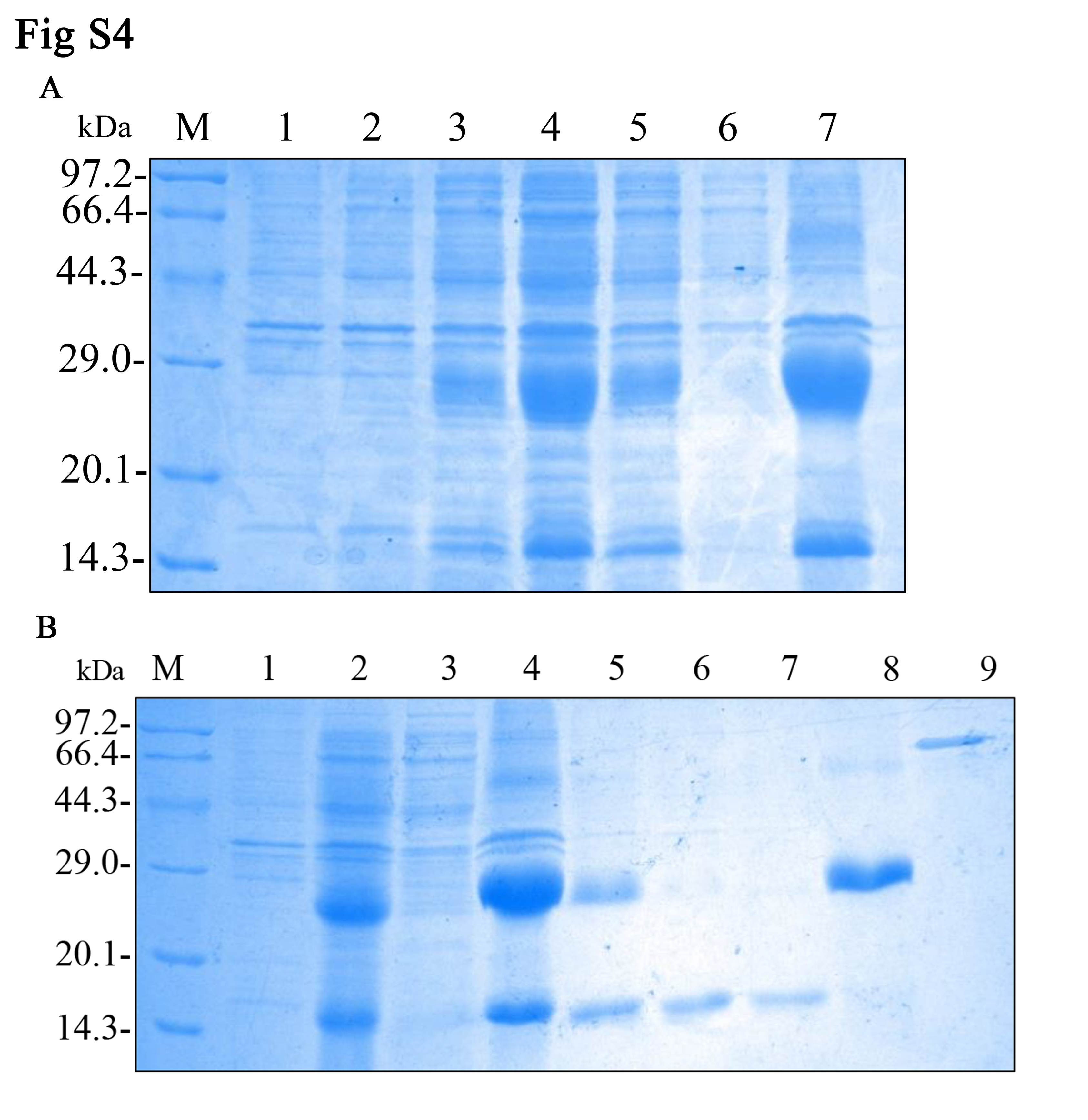
**


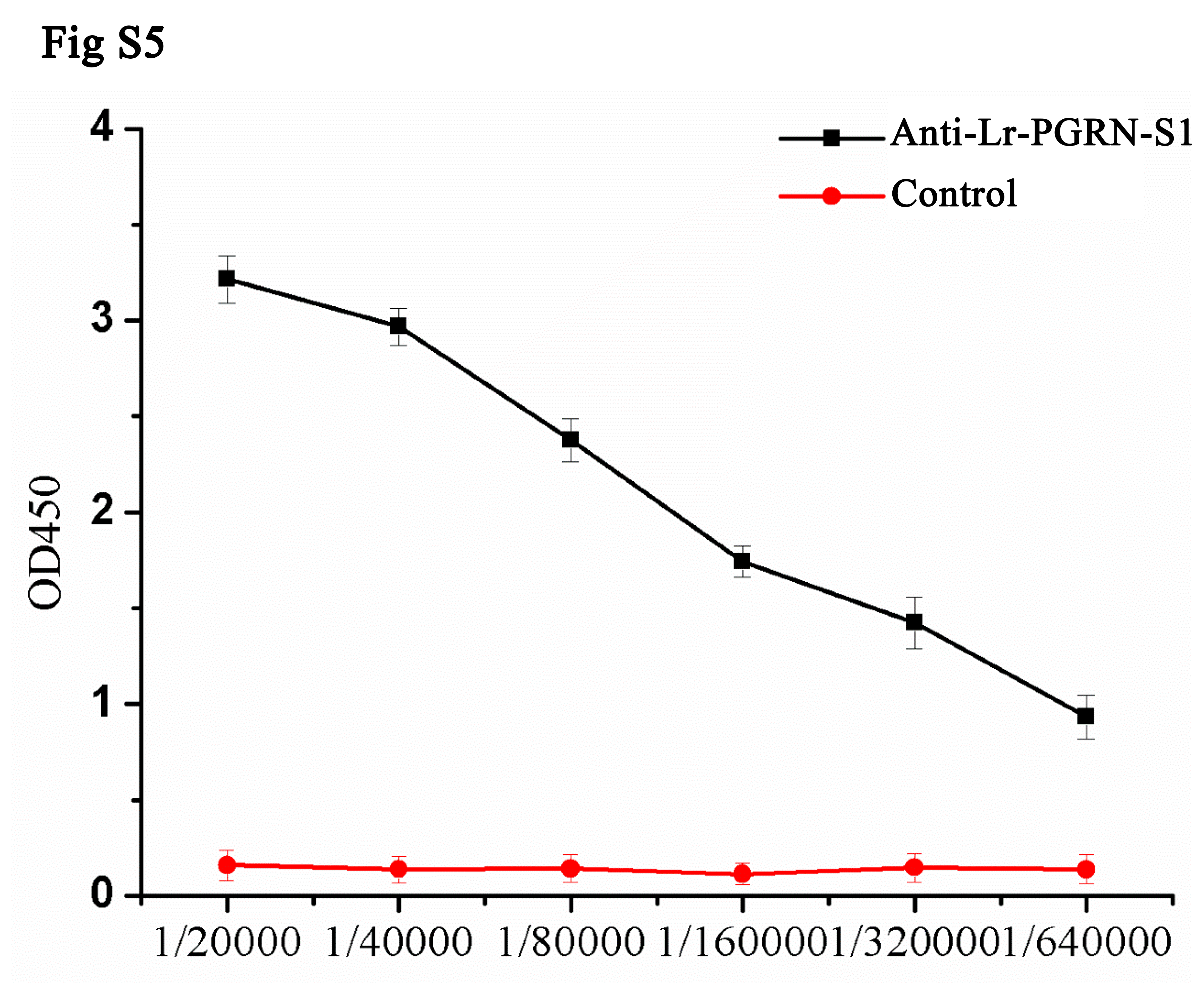


**
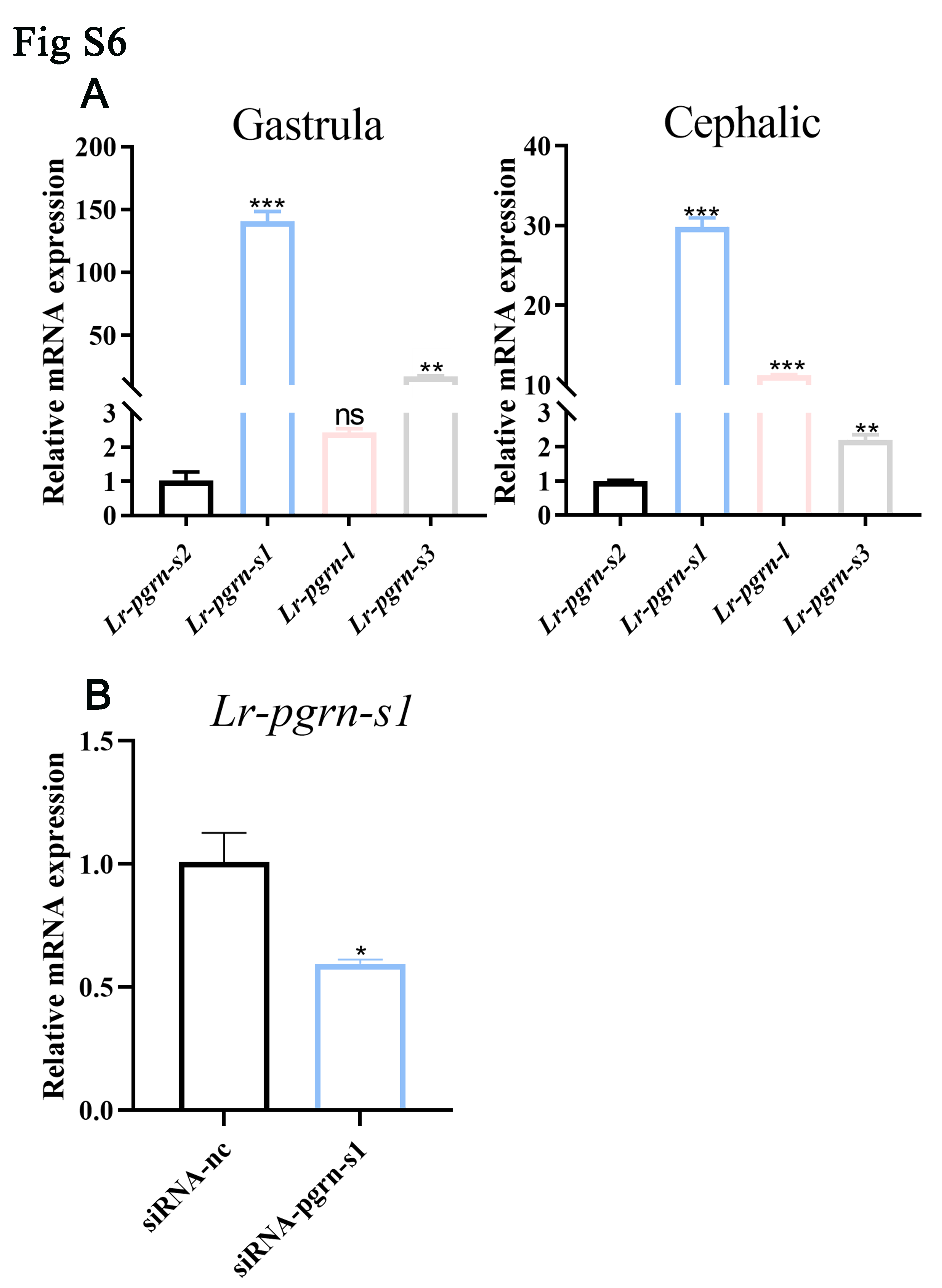
**





**

**

**Additional tables**

**Table S1. Primers used for PCR in this study.**

| Types of PCR | Primer name | Nucleotide sequence (5’-3’ ) |
| --- | --- | --- |
|  | *Lr-pgrn-l-F* | AGCAGGCAAAGTGAAAGCGGC |
|  | *Lr-pgrn-l -R* | CCAAGCACACAACAGACAGCG |
| *PCR* | *Lr-pgrn-s1-F* | TAGGGCAAGCAGTGGTATCAACG |
|  | *Lr-pgrn-s1-R* | GCGTTGTGATGTCTGCTTCCGTG |
|  | *Lr-pgrn-s2-F* | GGTGCTCTGAGTCGCTGATTT |
|  | *Lr-pgrn-s2-R* | GATGGGAGGTGGCTTTTGATG |
|  | *Lr-pgrn-s3-finner-F* | TTGATTAGAGGTGCCGATTAGT |
|  | *Lr-pgrn-s3-rinner-R* | GTATGAGATAGTGTGCGGGTGT |
|  | *Lr-pgrn-s3-fouter-F* | CCAAAAAAATGTCATCTCAGG |
|  | *Lr-pgrn-s3-router-R* | GAATGTCGTAAAACAACTCCC |
| **qRTPCR** | *Lr-pgrn-l-F* | ACCGTTTGCGATTTGGCTCA |
|  | *Lr-pgrn-l-R* | CCGTCCGCACACTGCTTATT |
|  | *Lr-pgrn-s1-F* | CACGATGGAATGAGACAAATGAA |
|  | *Lr-pgrn-s1-R* | CCATGATGTGTGGACCAGATG- |
|  | *Lr-pgrn-s2-F* | CTGCTGCTGATCGTGCCTTG |
|  | *Lr-pgrn-s2-R* | CCGGACGAATAGAGGCAGCA |
|  | *Lr-pgrn-s3-F* | ACTCTTGCTGCTGCTGGTG |
|  | *Lr-pgrn-s3-R* | AAACAGCAGTAAACGGTCAAGCC |
|  | *lrp6-F* | ACACGCTCTACTGGACCGACTG |
|  | *lrp6-R* | TGGGCGAGTAGATGTTGGAGAGG |
|  | *notch-F* | ACAGCAGCATCAGCAGCATCAG |
|  | *notch-F-R* | ATCTCGTTGGGCTTGTAGTCATTGG |
|  | *hes1-F* | CCGATCATGGAGAAGAGGAGGAGAG |
|  | *hes1-R* | CAACTTGGAGTGGCGAGAACTGTC |
|  | *crebbp-F* | GCGACGACTACATCTTCCACTGC |
|  | *Crebbp-R* | GATTCTCTCAGCCACTGCCTTGTC |
|  | *E2f4-F* | AGCTCATGTATTCGGACGTGTTCAC |
|  | *E2f4-R* | GTCACAAACGCCCTCAGTCTCG |
|  | *Ccnd1-F* | ACAATCTCAGTTCAGCAGCAGCAG |
|  | *Ccnd1-R* | CTCCTTCCTCCACCCGTGTCTC |
|  | *Dvl1-F* | CGAGGAGGAAACCCCGTATCTGG |
|  | *Dvl1-R* | GTTGAGCGCCGTCTTGAAGTCC |
|  | *Dll1-F* | GAACTCGCCGTGAACCTCTTAACC |
|  | *Dll1-R* | GCAGCCTTCTCCAGGATCATGTTG |
|  | *p38-F* | AACAACAGCAACAGCAGCAACAAC |
|  | *p38-R* | CACTCAATATCCGCCAGCGTCTC |
|  | *MAX-F* | CTAATGGCGGCTGTCGTCTGTC |
|  | *MAX-R* | GAAGCTGTCCTTGATGTGGTCTCTC |
|  | *TGFB2-F* | CACTCTTCAACAGCACCCGAGAC |
|  | *TGFB2-R* | TGTGGACCTCCTTGGCGTAGTAC |
|  | *Daxx-F* | AGATAACAGACGACACGGACTCCTC |
|  | *Daxx-R* | TCCACCTCACAAGTCTCGCTCTG |

**Table S2. Primers used for siRNA and vector linking in this study.**

| Purpose | Primer name | Nucleotide sequence (5’-3’ ) |
| --- | --- | --- |
| siRNA | *Lr-pgrn-s1-142-F* | GAUGGUCGAUUCUGCGUAUTT |
|  | *Lr-pgrn-s1-142-R* | AUACGCAGAAUCGACCAUCTT |
|  | *Lr-pgrn-s1-619-F* | GGAGCUCAAGUCGUGUACUTT |
|  | *Lr-pgrn-s1-619-R* | AGUACACGACUUGAGCUCCTT |
|  | *Lr-pgrn-s1-373-F* | GGAUCCACCUGCACCACAUTT |
|  | *Lr-pgrn-s1-373-R* | AUGUGGUGCAGGUGGAUCCTT |
| Eukaryotic expression primers | *Lr-pgrn-s1-F* | CGGACTCAGATCTCGAGCTCATGGGGGCTGTGTTCTCACTTGGGCT |
|  | *Lr-pgrn-s1-R* | AGAGGGGCGGATCCCGGGTCAATGATGATGATGATGATGGACACCCTCCA CAGCGGGCA |
